# Potassium Release From the Habenular Astrocytes Induces Depressive‐Like Behaviors in Mice

**DOI:** 10.1002/glia.24647

**Published:** 2024-11-29

**Authors:** Hidenori Aizawa, Miho Matsumata, Laura Ayaka Noguera Oishi, Fumie Nishimura, Deepa Kamath Kasaragod, Xintong Yao, Wanqin Tan, Tomomi Aida, Kohichi Tanaka

**Affiliations:** ^1^ Department of Neurobiology Graduate School of Biomedical and Health Sciences, Hiroshima University Hiroshima Japan; ^2^ Laboratory of Molecular Neuroscience Medical Research Institute, Tokyo Medical and Dental University Tokyo Japan

**Keywords:** astrocytes, depression, habenula, monoamines, optogenetics, potassium

## Abstract

The habenula has been implicated in psychiatric disorders such as depression, primarily because of its role in the modulation of the dopaminergic and serotonergic systems, which play a role in the pathophysiology of these disorders. Despite growing evidence supporting the role of the habenula in behavioral regulation, the process by which neural cells develop in the habenula remains elusive. Since the habenular anlage is found in the prosomere 2 domain expressing transcription factor Dbx1 in mouse embryos, we hypothesized that the Dbx1‐expressing prosomere domain is a source of astrocytes that modulate neuronal activity in the habenula. To address this, we examined the cell lineage generated from Dbx1‐expressing cells in male mice using tamoxifen‐inducible Cre recombinase under the control of the Dbx1 promoter. Perinatal induction of Cre activity labeled cells migrating radially from the ventricular zone to the pial side of the habenular anlage, and eventually showed astrocyte‐like morphology with expression of the marker protein, S100β, for mature astrocytes in the habenula of the adult mouse. Photostimulation of astrocytes expressing ChR2 released potassium ions into the extracellular space, which in turn excited the neurons with an increased firing rate in the lateral habenula. Finally, photostimulation of habenular astrocytes exacerbated depression‐like phenotypes with reduced locomotor activity, exaggerated despair behavior and impaired sucrose preference in open‐field, tail suspension and sucrose preference tests, respectively. These results indicated that the Dbx1‐expressing perinatal domain generated astrocytes that modulated neuronal activity via the regulation of extracellular potassium levels.

## Main Points

- Perinatal Dbx1‐expressing prosomere domain is a source of astrocytes in the habenula.
- Photostimulation of the astrocytes activates neurons in the habenula by increasing extracellular potassium ion leading to the depressive‐like behaviors.

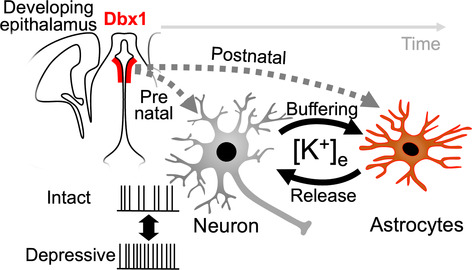

## 1 Introduction

The habenula is an evolutionarily‐conserved brain region that is part of the epithalamus. This structure has been implicated in psychiatric disorders, such as depression and addiction, primarily because of its role in the modulation of the dopaminergic and serotonergic systems, which play a role in the pathophysiology of these disorders (Aizawa and Zhu 2019). In particular, its involvement in depression is supported by accumulating evidence of altered activity of the lateral habenula in patients with depression and animal models of depression, while the experimental inhibition of habenular activity has an antidepressant effect in animal studies (Boulos, Darcq, and Kieffer 2017). These facts indicate that understanding the mechanisms by which neuronal activity in the habenula are regulated will provide deep insights into psychiatric disorders.

Despite growing evidence supporting the role of the habenula in behavioral regulation, the process by which neural cells develop in the habenula remains elusive. Forebrain development in mammals involves segmented domains that run longitudinally, known as prosomeres. Gene expression analysis has revealed that the habenular anlage is found primarily in the prosomere 2 (P2) domain in mouse embryos, expressing the transcription factor Dbx1 (Vue et al. 2007; Aizawa, Amo, and Okamoto 2011). Indeed, Dbx1‐expresing precursors were detected as early as embryonic day (E) 12.5 (E12.5) in the presumptive dorsal thalamus, from which the habenula develops. Dbx1‐expressing cells are found in the ventricular zone, from which the neurons are presumed to be born (Quina et al. 2009).

Recent studies have shown that astrocytes play a pivotal role in determining neuronal excitability (Bellot‐Saez et al. 2017). Since astrocytes in the cerebral cortex are generated locally from the ventricular zone of the same cortical anlage (Ge et al. 2012), we hypothesized that the Dbx1‐expressing prosomere domain is a source of astrocytes that modulate neuronal activity in the habenula. To address this, we traced the lineage of Dbx1‐expressing cells using tamoxifen‐inducible Cre recombinase under the control of the Dbx1 promoter.

Perinatal induction of Cre activity labeled cells migrating radially from the ventricular zone to the pial side of the habenular anlage, and eventually showed astrocyte‐like morphology with expression of the marker protein, S100β, for mature astrocytes. Photostimulation of astrocytes expressing ChR2 induced a significant increase in neuronal firing in the lateral habenula. Recordings with ion‐selective microelectrodes revealed that photostimulation released potassium ions into the extracellular space, which in turn excited neurons. Finally, photostimulation of habenular astrocytes exacerbated depressive‐like phenotypes with reduced locomotor activity, exaggerated despair behavior and impaired sucrose preference in open‐field, tail suspension and sucrose preference tests, respectively.

## 2 Methods

### 2.1 Animals

All experimental procedures were performed in accordance with the Animal Experiment (approval number A22‐139) and Recombinant DNA Experiment (approval number 2022‐90) plans approved by the Committee of Hiroshima University. Eight‐to‐twelve‐week‐old male wild‐type C57BL/6J (Japan SLC Inc., Shizuoka, Japan), *Dbx1* ^*CreERT2*^ (Hirata et al. 2009), Ai14 *Rosa* ^*tdTomato*^ (https://jaxmice.jax.org/strain/007914; RRID: IMSR_JAX:007914), and Ai32 *Rosa* ^*ChR2(H134R)‐EYFP*^ (https://jaxmice.jax.org/strain/024109.html; RRID: IMSR_JAX:024109) mice were used in the experiments. The mice were housed under a 12‐h light/dark (lights on from 8:00 AM to 8:00 PM) and had *ad libitum* access to water and food.

### 2.2 Birthdate‐Specific Labeling of Cells

For prenatal labeling, tamoxifen (T5648; Sigma–Aldrich Corp., St Louis, MO, USA) was dissolved in corn oil (20 mg/mL) and administered to pregnant female *Rosa* ^*ChR2(H134R)‐EYFP/ChR2(H134R)‐EYFP*^ mice crossed with male *Dbx1* ^*CreERT2/CreERT2*^ at a dose of 20 mg/kg. On E18.5, pups were retrieved by caesarian section and given to foster mothers (female Institute of Cancer Research strain mice). We also administered 5‐ethynyl‐2′‐deoxyuridine (EdU, 50 mg/kg; Click‐iT EdU Imaging Kits, C10339, Thermo Fisher Scientific Inc. Waltham, MA, USA) and bromodeoxyuridine (BrdU, 100 mg/kg; Cat. No. B5002, Sigma–Aldrich) to pregnant wild‐type female mice at E11.5 and E13.5, respectively.

For postnatal labeling, *Dbx1* ^*CreERT2/wt*^; *Rosa* ^*ChR2(H134R)‐EYFP*/*wt*^ mice received 5 μL of tamoxifen solution (prepared as above) at postnatal day (P) 0–2 (P0–P2) via intraperitoneal injection using a microliter syringe (Cat. no. 80500; Hamilton Company, Reno, NV, USA).

### 2.3 Histology

For immunohistochemistry, animals were sacrificed using an overdose of ketamine and fixed by transcardiac perfusion with 4% paraformaldehyde in 0.1 M phosphate buffered saline (PBS) (pH 7.4). After fixation overnight, 75‐μm‐thick sections (150 μm apart) were cut using a vibratome (DTK‐1000N; Dosaka EM Co. Ltd., Kyoto, Japan) and processed for immunohistochemistry.

Monoclonal mouse anti‐S100b (1:500; Cat# S2532, RRID: AB_477499, Sigma–Aldrich), polyclonal rabbit anti‐GFAP (1:1000, Cat# Z0334, RRID: AB_10013382, Agilent Technologies, Santa Clara, CA, USA), polyclonal rabbit anti‐Sox2 (1:500; Cat# AB5603, RRID: AB_2286686, Sigma–Aldrich), polyclonal rabbit anti‐Olig2 (1:500; Cat# 18953, RRID: AB_1630817, Immuno‐Biological Laboratories Co. Ltd., Fujioka, Japan), monoclonal rabbit anti‐c‐Fos antibodies (1:500; Cat# 2250, RRID: AB_2247211, Cell Signaling Technology Inc. Danvers, MA, USA), goat anti‐rabbit IgG‐AlexaFluor488 (1:500; Cat# A‐11034, RRID: AB_2576217, Invitrogen, Carlsbad, CA, USA), donkey anti‐mouse IgG‐AlexaFluor488 (1:500; Cat# ab150105, RRID: AB_2732856, Abcam, Cambridge, UK), and donkey anti‐rabbit IgG‐AlexaFluor594 (1:500; Cat# 711‐585‐152, RRID: AB_2340621, Jackson ImmunoResearch Laboratories Inc. West Grove, PA, USA) were used for fluorescent immunostaining.

For birthdate‐specific labeling, EdU was visualized using the procedure provided by the manufacturer, and then a signal for BrdU was developed according to previously described methods (Soma et al. 2009). Briefly, the sections were treated with 2 N HCl in saline for 1 h, followed by washing with PBS. Monoclonal mouse anti‐BrdU (1:100; Cat# B8434; RRID: AB_476811, Sigma–Aldrich) and donkey anti‐mouse IgG‐AlexaFluor488 (1:500; Cat# ab150105, RRID: AB_2732856, Abcam) antibodies were used for BrdU detection. Some sections were stained with 4′,6‐diamidino‐2‐phenylindole.

The sections were imaged using an electron‐multiplying charge‐coupled device (CCD) camera (iXon Ultra 897, Andor Technology Ltd., Belfast, UK) attached to a confocal microscope (Revolution XD, Andor Technology Ltd.) under the control of μManager (Edelstein et al. 2010).

Quantification of the area in the habenula expressing ChR2‐EYFP was performed using Fiji software (Schindelin et al. 2012). Briefly, we binarized the image following background subtraction and calculated the percentage of the area with a signal in the region of interest. For counting the number of cells expressing c‐Fos protein, the “Analyze Particles” function of Fiji was applied for quantification. The number of cells were counted manually with the help of Cell Counter plugins available in Fiji.

### 2.4 Implantation of Optical Cannula for Optogenetics

Mice were deeply anesthetized with a mixture of ketamine (90 mg/kg) and xylazine (10 mg/kg) and immobilized in a stereotaxic apparatus (SR6N, Narishige, Tokyo, Japan). Burr holes were made to implant the optical cannula targeting to the bilateral lateral habenula (1.7 mm posterior and 0.4 mm lateral to the bregma; 1.9 mm deep from the pial surface) with dental adhesive (super‐bond C&B set, SunMedical, Moriyama, Japan) and dental cement (UNIFAST2, GC corporation, Tokyo, Japan). Mice were kept at least 4 days at home cage for recovery before we performed behavioral tests.

### 2.5 Behavioral Tests

All behavioral tests were performed after the mice were habituated to the test room for at least 1 h. Mice received blue light (460 nm for the test group; Optogenetics‐LED‐Blue, Prizmatix, Holon, Israel) or yellow light (595 nm for the control group; Optogenetics‐LED‐Yellow, Prizmatix) for 20 min with 2‐s on/2 s off cycles in their home cage before (open‐field and tail suspension tests) or during tests (sucrose preference test). The blue and yellow light intensities at the fiber tip were 4.0 and 1.8 mW/mm^2^, respectively.

#### 2.5.1 Open‐Field Test

An open‐field arena (50 × 50 × 40 cm^3^) under 70 lx illumination was used for the test. Mice were placed at the corner of the arena, and their movement was recorded for 10 min using a CCD camera (MK‐0323E, Mintron Enterprise Co. Ltd., New Taipei City) at 60 fps. The video images were subsequently processed for analysis using ImageOF (Mouse Phenotype Database; http://www.mouse‐phenotype.org/software.html) based on the ImageJ program (https://imagej.nih.gov/ij/) (Schneider, Rasband, and Eliceiri 2012).

#### 2.5.2 Tail Suspension Test

The tail of each mouse was suspended by attaching it to a smooth Plexiglas plate under the 70 lx illumination and the mouse was hung from the roof of a sound attenuation box. The animal movements were recorded for 6 min using a CCD camera. Subsequently, the duration of the animal's immobility was scored and analyzed using ImageFZ software (Mouse Phenotype Database). The criteria for the immobile and struggling sessions were immobile and struggling for more than 3 s each.

#### 2.5.3 Sucrose Preference Test

Sucrose preference test was conducted using 2% sucrose and tap water as described previously with slight modification (Zhang et al. 2024). Briefly, mice were singly‐housed and habituated to the home cage with 2 bottles of tap water for 2 days, then 2 bottles of 2% sucrose for additional 2 days. Following water deprivation for 24 h, mice were exposed to one bottle of 2% sucrose and another bottle of tap water for 2 h during light on period. The position of these bottles was exchanged 1 h after the onset of test. Sucrose preference in percentage was calculated as (total consumption of 2% sucrose)/(total consumption of both 2% sucrose and tap water) × 100.

### 2.6 Potassium‐Selective Microelectrode Fabrication

Potassium‐selective microelectrodes were fabricated according to a previously described procedure, with slight modifications (Octeau et al. 2018). Briefly, a glass tube (B150‐86‐10, Sutter Instrument Company, Novato, CA, USA) was pulled and silanized with 5% dichlorodimethylsilane (D0358, Tokyo Chemical Industry Co. Ltd., Tokyo, Japan), followed by baking at 200°C. The tip of the glass pipette was filled with a valinomycin‐based potassium ionophore (potassium ionophore I‐cocktail B, Cat. # 99373‐0.1ML‐F, Sigma–Aldrich), and then back‐filled with 100 mM KCl. Potential was amplified with an amplifier (gain, ×50; MultiClamp700A, Axon Instruments Inc. Seattle, WA, USA) and sampled at 1000 Hz (digidata1322A, Axon Instruments Inc.). We applied three solutions with different KCl concentration: 1 mM KCl (25 mM 4‐[2‐hydroxyethyl]‐1‐piperazineethanesulfonic acid [HEPES], 1 mM KCl, and 150 mM NaCl), 2.5 mM KCl (artificial cerebrospinal fluid [ACSF; 125 mM NaCl, 2.5 mM KCl, 1.25 mM NaH_2_PO_4_, 26 mM NaHCO_3_, 20 mM D‐glucose, 1 mM MgSO_4_, and 2 mM CaCl_2_]) and 10 mM KCl (25 mM HEPES, 10 mM KCl, and 150 mM NaCl) at room temperature to an electrode in the recording chamber (CK‐1, Narishige Scientific Instrument Lab., Tokyo, Japan) via a superfusion system driven by a peristaltic pump (Minipuls3, Gilson Co. Inc., Middleton, WI, USA), at a flow rate of 2 mL/min. We used ACSF for 2.5 mM KCl calibration, since it provided the potential comparable with 2.5 mM KCl prepared in HEPES‐based buffer.

The potential response of the potassium‐selective microelectrode was assessed by calculating a linear slope of the potential plotted against the natural logarithm of the potassium concentration with high correlation (*R* ^2^, 0.99 ± 0.0010 in Pearson's correlation, *N* = 14 electrodes). We observed a mean slope of 59.4 ± 0.26 mV/log[K^+^] (*N* = 14 electrodes) compatible with the slope predicted by the Nernst equation at room temperature (58.4 mV/log[K^+^]) (Octeau et al. 2018).

### 2.7 Measurement of the Extracellular Potassium Concentration [K^+^]_e_ In Vitro

*Dbx1* ^*CreERT/wt*^; *Rosa* ^*ChR2(H134R)‐EYFP/wt*^ mice without (control group) or with tamoxifen injection at P0–P2 (test group) were deeply anesthetized with 2%–3% isoflurane, perfused with cold ACSF (see above), and decapitated quickly. We cut 300 μm‐thick sections from the dissected brain in the chamber filled with cutting solution (2.5 mM KCl, 1.25 mM NaH_2_PO_4_, 30 mM NaHCO_3_, 20 mM HEPES, 92 mM N‐methyl‐D‐glucamine, 2 mM thiourea, 5 mM sodium L‐ascorbate, 3 mM sodium pyruvate, 25 mM D‐glucose, 10 mM MgSO_4_, and 0.5 mM CaCl_2_). Sections were initially recovered in the recovery solution (92 mM NaCl, 2.5 mM KCl, 1.25 mM NaH_2_PO_4_, 30 mM NaHCO_3_, 20 mM HEPES, 2 mM thiourea, 5 mM sodium L‐ascorbate, 3 mM sodium pyruvate, 25 mM D‐glucose, 2 mM MgSO_4_, and 2 mM CaCl_2_), then transferred to the maintenance chamber in ACSF with continuous carbogenation.

A potassium‐selective microelectrode was placed on the lateral habenula of the slice, which was transferred to the recording chamber perfused with ACSF at a flow rate of 2 mL/min. To block the action potential of neurons or inward rectifier potassium channel (Kir), tetrodotoxin (500 nM in ACSF; Cat. #206‐11071, FUJIFILM Wako Pure Chemical Corp., Osaka, Japan) or barium chloride dihydrate (BaCl_2_, 100 μM applied for 15 min, Cat. #029‐00175, FUJIFILM Wako Pure Chemical Corp., Osaka, Japan) (Cui et al. 2018) was applied in some experiments, respectively. The potential was recorded as in the calibration by applying various widths (ranging from 0.5 to 5 s) of pulses of 470 nm light (5 mW/mm^2^) every 180 s through an optical fiber (M28L, Thorlabs Inc., Newton, NJ, USA) coupled to a light source (M470L3, Thorlabs Inc.). The acquired data were analyzed using a custom‐written script running in MATLAB (RRID:SCR_001622, MathWorks Inc., Natick, MA, USA).

### 2.8 In Vivo Electrophysiology

*Dbx1* ^*CreERT/wt*^; *Rosa* ^*ChR2(H134R)‐EYFP/wt*^ mice were anesthetized by intraperitoneal injection of urethane (1.5 g/kg) and placed in a stereotaxic frame (SR‐6M, Narishige Scientific Instrument Lab.). The body temperature was monitored and maintained using a heating pad (BWT‐100A; BioResearch Center Co. Ltd., Nagoya, Japan). An optrode was fabricated by attaching a tetrode made of four twisted Ni‐Cr wires (12 μm in diameter; Precision Fine Tetrode Wire with Easy Bond XTC, Sandvik AB, Stockholm, Sweden) to the optical fiber (200 μm, 0.22 NA, Cat# M25L; Thorlabs Inc.). We inserted the optrode into the lateral habenula (1.7 mm posterior and 0.5 mm lateral to the bregma, 2.0–2.6 mm deep from the pial surface) and recorded the extracellular unit activity sampled at 20,000 Hz (RHD Recording System, Intan Technologies, Los Angeles, CA, USA) with application of a 2 s‐pulse of 470 nm light (65 mW/mm^2^, M470L3, Thorlabs Inc., Newton, NJ) at 0.25 Hz for 20 min.

The acquired data were processed to isolate spike events using EToS (https://etos.sourceforge.net/) (Takekawa, Isomura, and Fukai 2012) and sorted to refine single‐neuron clusters using Klusters and Neuroscope (Hazan, Zugaro, and Buzsáki 2006). The peri‐stimulus time histogram was calculated using the Cell Explorer (Petersen et al. 2021) in MATLAB (MathWorks Inc.).

### 2.9 Statistical Analysis

The mean difference between groups was analyzed by a one‐tailed Welch's *t‐*test with effect size for comparison between two groups and one‐way ANOVA, followed by Tukey's *post hoc* test for multiple comparisons for data with three or more groups. Analyses were performed using jamovi 2.3 (The jamovi project 2023, https://www.jamovi.org). For in vivo electrophysiology, the difference in the mean firing rate before and after illumination was analyzed using the Kolmogorov–Smirnov test in MATLAB (MathWorks Inc.). The level of significance was defined as *p* < 0.05, and all values were indicated as mean ± SEM.

### 2.10 Code Accessibility

Custom MATLAB codes used for analysis of extracellular potassium are available upon request.

## 3 Results

### 3.1 Generation of Non‐neuronal Cells From the Dbx1‐Positive Domain in the Ventricular Zone

**FIGURE 1:**
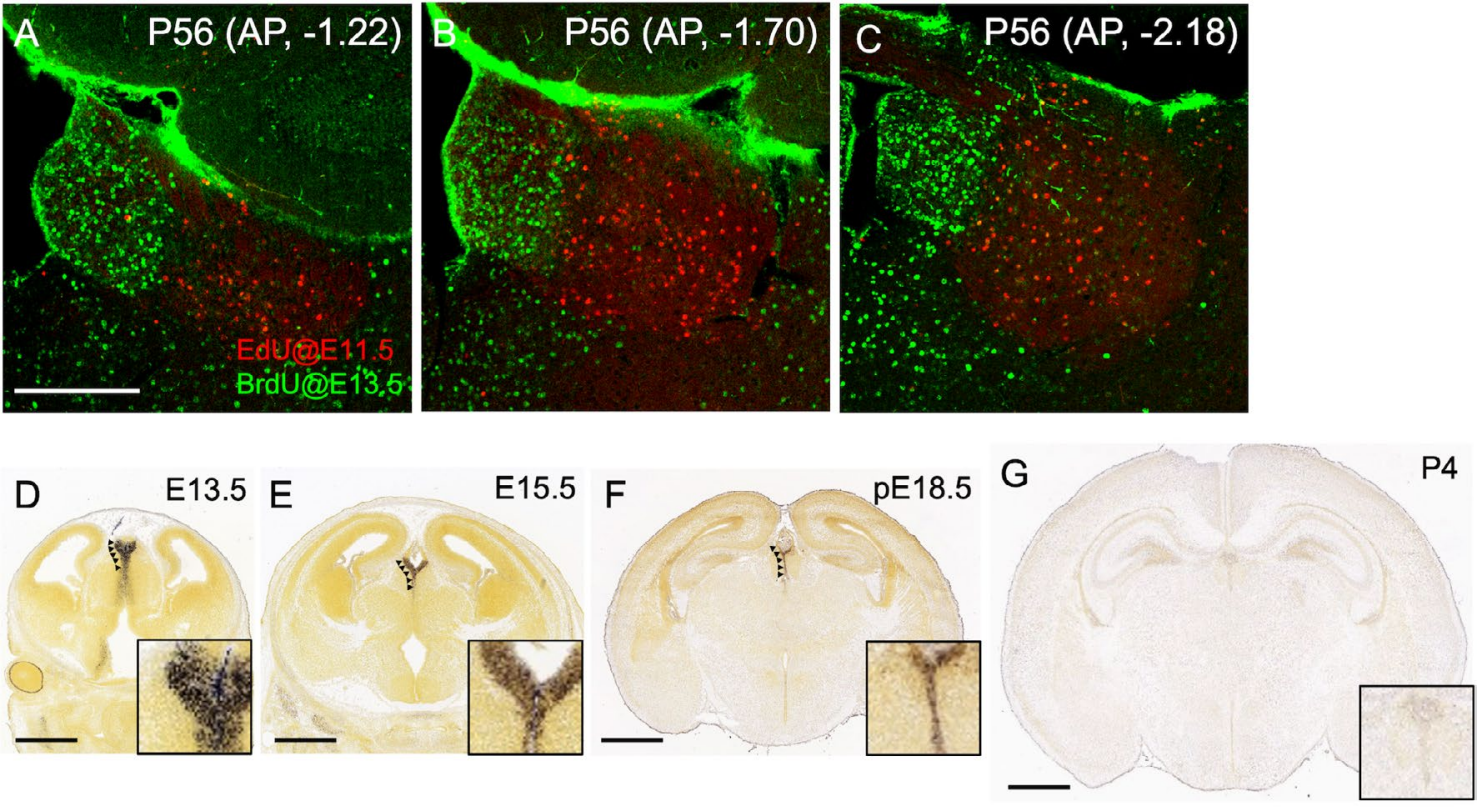
Dbx1 is expressed in the epithalamic region of the perinatal brain. (A–C) Coronal sections of the habenula at the level of 1.22 (A), 1.70 (B), and 2.18 mm (C) posterior to the bregma of an adult mouse which received prenatal administrations of 5‐ethynyl‐2′‐deoxyuridine (red) and bromodeoxyuridine (green) at E11.5 and E13.5. (D–G) Coronal sections of the mouse brain showing Dbx1 mRNA expression (purple) at the ventricular zone in the epithalamus (arrowheads) at E13.5 (D), E15.5 (E), presumptive E18.8 (pE18.5 in F), and P4 (G) Images are derived from Allen Institute for Brain Science (https://developingmouse.brain‐map.org/). Scale bars, 250 μm (A, applies to panels B and C) and 1 mm (D–G).

We first examined the birthdate of the neurons in the habenula by BrdU and EdU pulse labeling method (Shibui et al. 1989). These uridine derivatives incorporated at mitotic phase in cell cycle is supposed to be kept at high concentration without further cell division in neuron, while it is to be lost significantly in the non‐neuronal cells which further proliferates with multiple cell divisions (Miller and Nowakowski 1988). We observed that cells born at E11.5 gave rise to the neurons in the lateral habenula (red in Figure 1A–C), while those born at E13.5 gave rise to the neurons in the medial habenula (green Figure 1A–C) along the entire anterior–posterior axis in the P56 mice. On the other hand, pulse labeling at E18.5 did not label any neurons in P56 mice in accordance with a previously published report using thymidine‐H^3^ (Angevine, 1970) (data not shown), suggesting that the non‐neuronal cells such as astrocytes is likely to exit cell cycle and differentiate after E18.5 as in the other brain regions such as cerebral cortex (Rowitch and Kriegstein 2010).

To explore the origin of those glia in the habenula, we focused on *Dbx1* gene which was reported to express in the caudal and dorsal part of the thalamus including the habenular but not pretectal anlage (Vue et al. 2007) and the hypothalamic areas (Sokolowski et al. 2016). Indeed, the Developing Mouse Brain Atlas (https://developingmouse.brain‐map.org/) revealed that *Dbx1* mRNA was consistently found in the ventricular zone of the entire epithalamus in the prenatal thalamus from E13.5 to presumptive E18.5 (Figure 1D–F), while none of those regions expressed *Dbx1* mRNA at P4 (Figure 1G).

**FIGURE 2:**
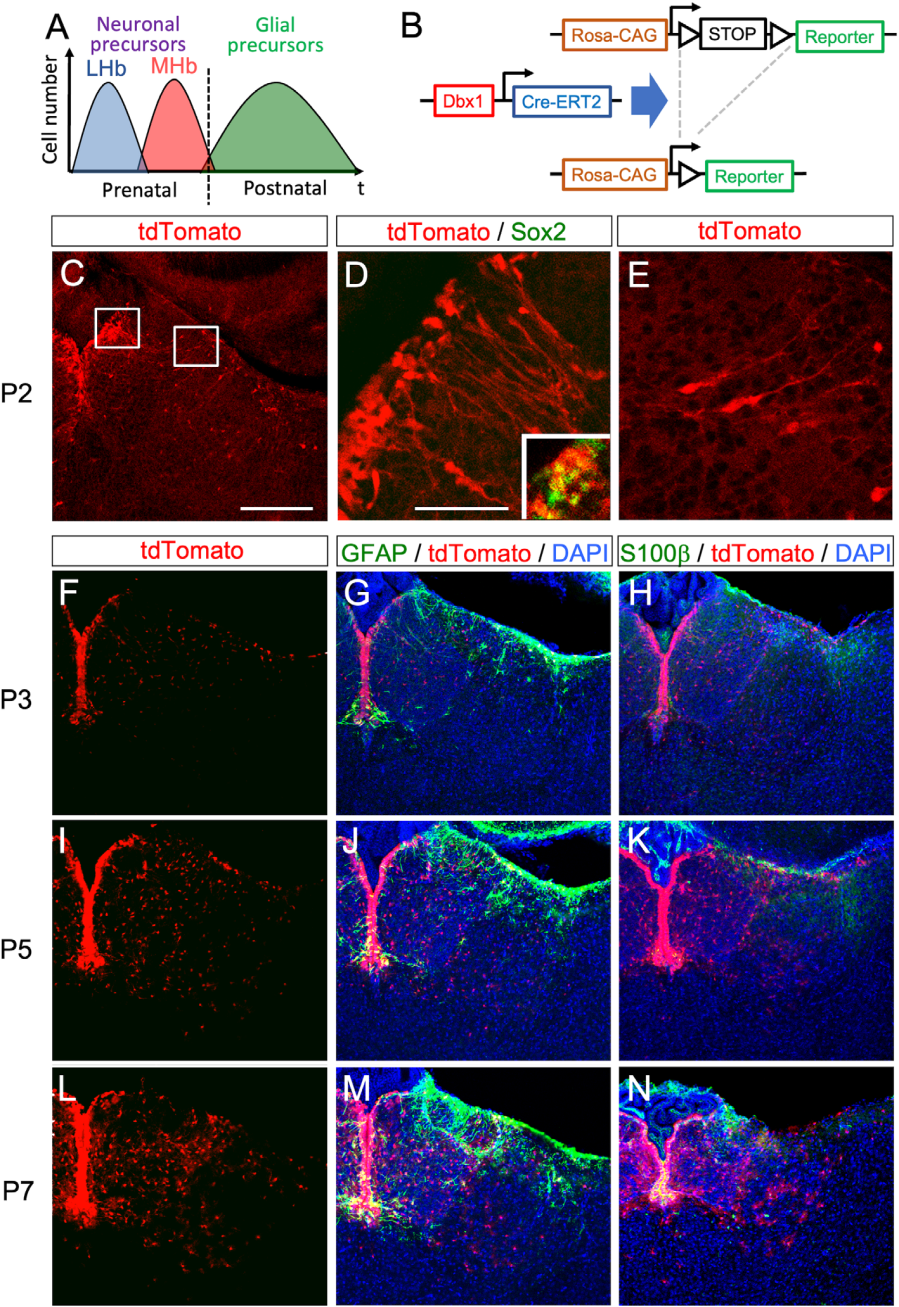
Cells born postnatally migrate from the Dbx1‐positive ventricular zone of the epithalamic anlage. (A) A schematic diagram for generation of neuronal (lateral habenula, LHb, blue; medial habenula, MHb, red) and glial precursors (green) during prenatal and postnatal periods, respectively. (B) A schematic diagram showing recombination of the Rosa‐CAG‐loxP‐STOP‐loxP‐Reporter cassette in the presence of Dbx1‐CreERT2. Open triangles, loxP. Rosa‐CAG, chicken beta‐actin promoter in the Rosa26 locus. (C–E) Coronal sections of the habenula of a *Dbx1* ^*CreERT2/wt*^; *Rosa* ^*tdTomato/wt*^ mouse mice at P2 with tamoxifen injection at P0 showing the distribution of cells expressing tdTomato (red in C–E) and Sox2 (green in inset of D). Panels D and E are a magnified view of the left and right boxed area in panel C. (F–N) Coronal sections of the habenula of P3 (F–H), P5 (I–K), and P7 (L–N) *Dbx1* ^*CreERT2/wt*^; *Rosa* ^*tdTomato/wt*^ mice showing the distribution of the cells expressing tdTomato (red), GFAP (green in G, J, M), and S100β (green in H, K, N). Scale bars, 200 μm (C, applies to panels F–N) and 50 μm (D, applies to panel E).

These facts led us to hypothesize that the Dbx1‐positive domain of the diencephalic ventricular zone generates the glia in the habenula after E18.5 (Figure 2A).

### 3.2 Dbx1‐Positive Domain Postnatally Gives Rise to Habenular Astrocytes

To address the hypothesis described above, we generated *Dbx1* ^*CreERT/wt*^; *Rosa* ^*tdTomato/wt*^ in which tdTomato, as a reporter, was expressed when Cre‐ERT2 was translocated into the nucleus of the cells at the Dbx1‐positive ventricular zone upon tamoxifen administration (Figure 2B). We first injected tamoxifen into newborn *Dbx1* ^*CreERT/wt*^; *Rosa* ^*tdTomato/wt*^ mice at P0 and checked the distribution of labeled cells at P2 (Figure 2C–E). The majority of the cells labeled with tdTomato were found in the area next to the ventricle (Figure 2C,D) and expressed the neural stem cell marker, Sox2 (green in the inset of Figure 2D). Some cells were scattered in the habenular anlage, leaving the ventricular area to reach the pial surface. They exhibited a long cellular process toward the pial side, which was asymmetrically reminiscent of migratory cells and their leading processes (Figure 2E). Further tracing of the subsequent migration of these labeled cells revealed that they increased in number and were distributed more widely in the habenular areas at P3 (Figure 2F) and P5 (Figure 2I). Intriguingly, cells labeled with tdTomato migrated radially from the ventricular zone, but remained in the habenular areas without migrating across the lateral margin of the lateral habenula at P7 (Figure 2L), although minor cells migrated ventrally to the paraventricular (PV) nucleus. During the early postnatal period from P3–P7, GFAP‐ (Figure 2G,J,M) and S100β‐expressing cells (Figure 2H,K,N) were primarily limited to cells in the ventricular zone and stria medullaris.

**FIGURE 3:**
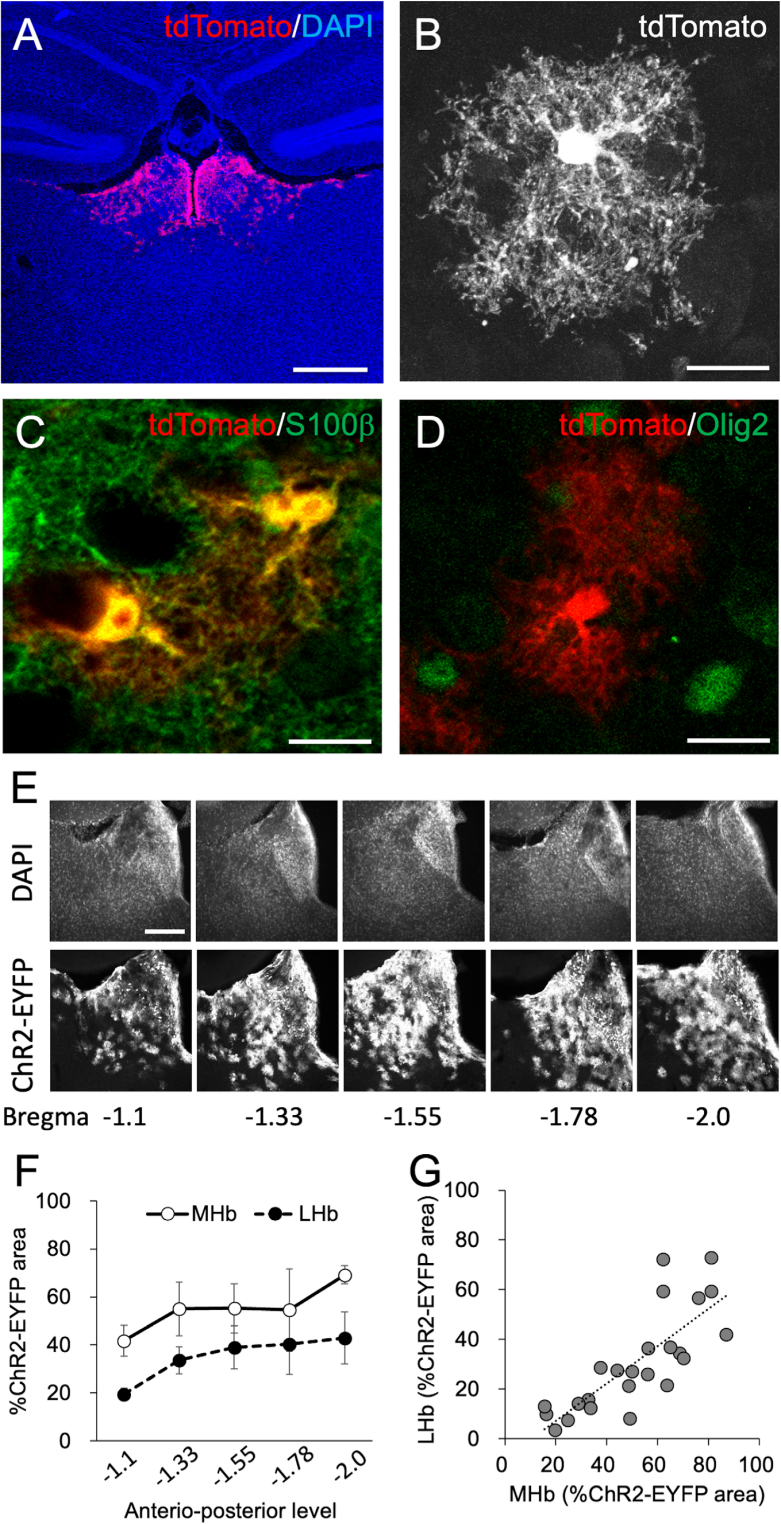
Dbx1‐derived cells give rise to the S100β–expressing astrocytes in the habenula. (A–D) Coronal sections of P21 of a *Dbx1* ^*CreERT2/wt*^; *Rosa* ^*tdTomato/wt*^ mouse with tamoxifen injection at P0 showing tdTomato (red in A, C, D; white in B), S100b (green in C), and Olig2 (green in D). (E) A montage of coronal sections of the habenula at the various levels of the anterior–posterior level showing nuclear staining with 4′,6‐diamidino‐2‐phenylindole (DAPI) (top) and ChR2‐EYFP (bottom). (F) A scatter plot of the proportion of the area in the medial (MHb, abscissa) and lateral habenula (LHb, ordinate) expressing ChR2‐EYFP. Dashed line and formulae represent lines and the results of linear regression analysis, respectively. Data are presented as mean ± standard error of mean (SEM). (G) A line plot of proportional area expressing ChR2‐EYFP in the MHb (open circles with solid line) and LHb (closed circles with dashed line) at various level of anterior–posterior level of the habenula. Scale bars, 500 μm (A), 20 μm (B), 10 μm (C, D), and 100 μm (E).

These Dbx1 domain‐derived cells remained in the habenular area at P21 and later (Figure 3A) and showed astrocyte‐like morphology with fine and short cellular processes, but not thick and long dendrites and axons (Figure 3B). Histological analysis using astrocyte (S100β) and oligodendrocyte markers (Olig2) revealed that 90.6% and 87.3% of these cells co‐expressed S100β (*N* = 350 and 267 cells for the medial and lateral habenula, respectively) (Figure 3C), while the 3.2% and 8.8% of them co‐expressed Olig2 in the medial and lateral habenula, respectively (*N* = 375 and 422 cells for the medial and lateral habenula, respectively) (Figure 3D). Unlike their co‐expression of these glial markers, we did not observe any of Dbx1‐CreERT2; Rosa‐tdTomato‐positive cells co‐expressed a neuronal marker NeuN (*N* = 64 and 103 cells for the medial and lateral habenula, respectively), suggesting that Dbx1 domain‐derived cells gave rise to non‐neuronal cells in the habenula.

Since we observed the same distribution of labeled cells as above when we injected tamoxifen from P0 to P2, but not after P3, Dbx1 domain‐derived cells in the ventricular zone seemed to continue generating habenular astrocytes during the early neonatal period. The distribution of these cells was further examined by labeling them with the membrane‐tethered fluorescent protein ChR2‐EYFP in *Dbx1* ^*CreERT/wt*^; *Rosa* ^*ChR2(H134R)‐EYFP/wt*^ (Figure 3E). The ChR2‐EYFP expressing area covered the habenula posteriorly, with a wider distribution in the medial habenula (open circles in Figure 3F) than in the lateral habenula (closed circles in Figure 3F; *F*_1,30_ = 9.739, *p* = 0.004). The proportion of the area covered by Dbx1 domain‐derived astrocytes was comparable between the medial and lateral habenula, irrespective of the anteroposterior level (Figure 3G, Pearson's *r* = 0.788, *p* < 0.001). We also observed the ChR2‐EYFP expressing cells in the posterior thalamus, hypothalamus and mammillary body in accordance with Dbx1 mRNA expression in the ventricular zone at E18.5 as described above (Figure S1).

Collectively, these results indicated that the Dbx1‐positive domain postnatally gives rise to astrocytes that cover the entire habenula.

### 3.3 Dbx1‐Derived Habenular Astrocytes Modulate Neuronal Activity

To address the functional role of astrocytes in the modulation of neural activity in the habenula, we expressed ChR2 in cells derived from the Dbx1‐expressing domain using adult *Dbx1* ^*CreERT/wt*^; *Rosa* ^*ChR2(H134R)‐EYFP/wt*^ mice injected with tamoxifen at P0–P2.

**FIGURE 4:**
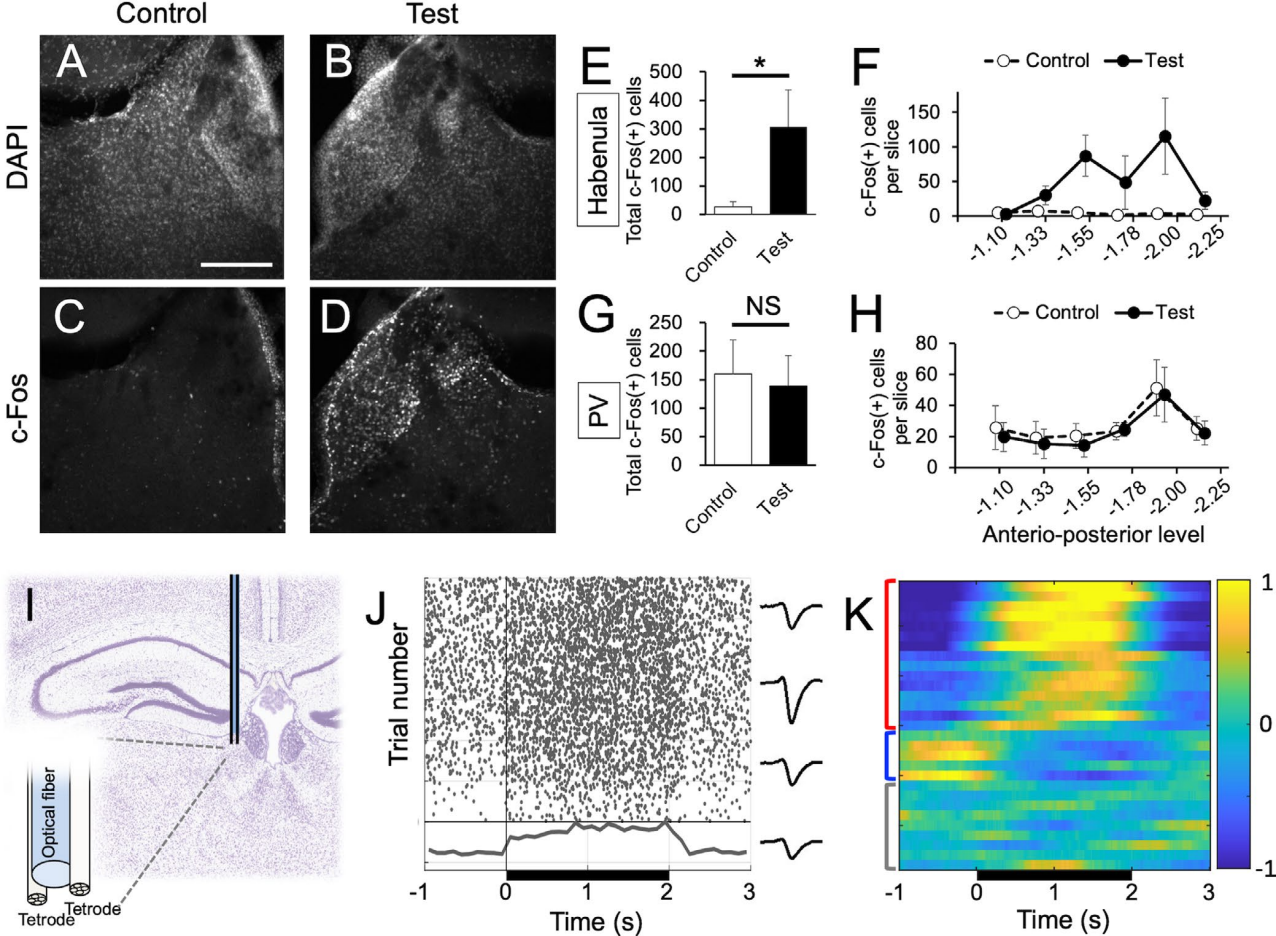
Photostimulation of the ChR2‐expressing astrocytes leads to the habenular activation with increased firing rate of the neurons. (A–D) Coronal sections of the habenula without (A, C) and with photostimulation (B, D) showing the nucleus stained with DAPI (A, B) and c‐Fos protein expression (C, D). (E, G) Bar graphs of the total number of c‐Fos‐positive cells in the habenula (E) and paraventricular (PV) thalamic nucleus (G) on the control (white) and test side with photostimulation (black). (F, H) Line plots of the c‐Fos‐expressing cells per slice at a various anterior–posterior level of the habenula on the control (open circles and dashed line) and test side (closed circles and solid line). Values of abscissa are the distance from the bregma. (I) A coronal section of the adult mouse brain showing a location of the optrode (schematic illustration in the inset) in the lateral habenula. (J) A raster plot (left top), peri‐stimulus time histogram (PSTH; left bottom) and waveform (right) of a representative unit activity of the lateral habenular neuron in response to photostimulation for 2 s. (K) Normalized PSTH of the spikes responding to the photostimulation. Brackets with red, blue, and gray colors indicate the cells responded positively, negatively, and not significantly, respectively. Black bars at the bottom of panels J and K are periods with illumination. Data are presented as mean ± SEM. *, *p* < 0.05. NS, not significant.

We first examined the effect of photostimulation of habenular astrocytes using c‐Fos protein expression as a marker of neural activation. Unilateral photostimulation of ChR2‐expressing astrocytes resulted in an increase in cells expressing c‐Fos in the entire habenula on the targeted side compared with the control side without photostimulation (Figure 4A–D). As ChR2‐EYFP was also partially expressed in the PV thalamic nucleus, ventromedial to the habenula (Figure 4E), we tested whether photostimulation of the habenula also modulated the activity of PV cells by examining c‐Fos‐positive cells. The results showed that photostimulation significantly increased the total number of c‐Fos‐positive cells in the habenula (Figure 4E, *t*_5.02_ = −2.69, *p* = 0.022, Cohen's *d* = −1.55, *N* = 6 mice for each group), whereas the total number of c‐Fos‐positive cells was comparable between the stimulated and non‐stimulated sides in the PV (Figure 4G, *t*_7.92_ = 0.270, *p* = 0.603, Cohen's *d* = 0.171, *N* = 5 mice for each group). Specifically, photostimulation significantly increased c‐Fos expression in the middle and posterior habenula (Figure 4F), while it did not affect c‐Fos expression, irrespective of the anterior–posterior level of the PV (Figure 4H).

Next, we electrophysiologically examined the effect of astrocyte photostimulation on the ongoing activity of neurons using an optrode (Figure 4I). Upon illumination of the lateral habenula with 460 nm light for 2 s, extracellular unit recording revealed a significant increase in the firing rate of neurons in the illuminated area (Figure 4J). Indeed, the activation of neuronal firing persisted in accordance with the prolonged duration of light stimulation. Intriguingly, the lateral habenular neurons responded to light stimulation with a gradual increase in their firing rate, which lasted even after light application ended (Figure 4J). Population analysis revealed that 69.0% of the recorded cells significantly changed their firing rates in response to illumination (*N* = 29). The majority of these neurons responded positively (15 out of 20 neurons, red brackets in Figure 4K), whereas the rest were reduced (blue brackets in Figure 4K) or did not change their firing rate significantly in response to photostimulation (gray brackets in Figure 4K).

These results implied that photostimulation of ChR2‐expressing astrocytes releases a mediator that acts on neurons to modulate their firing activity in the habenula.

### 3.4 Photoactivation of ChR2‐Expressing Astrocytes Releases Potassium to Modulate Neuronal Activity in the Habenula

**FIGURE 5:**
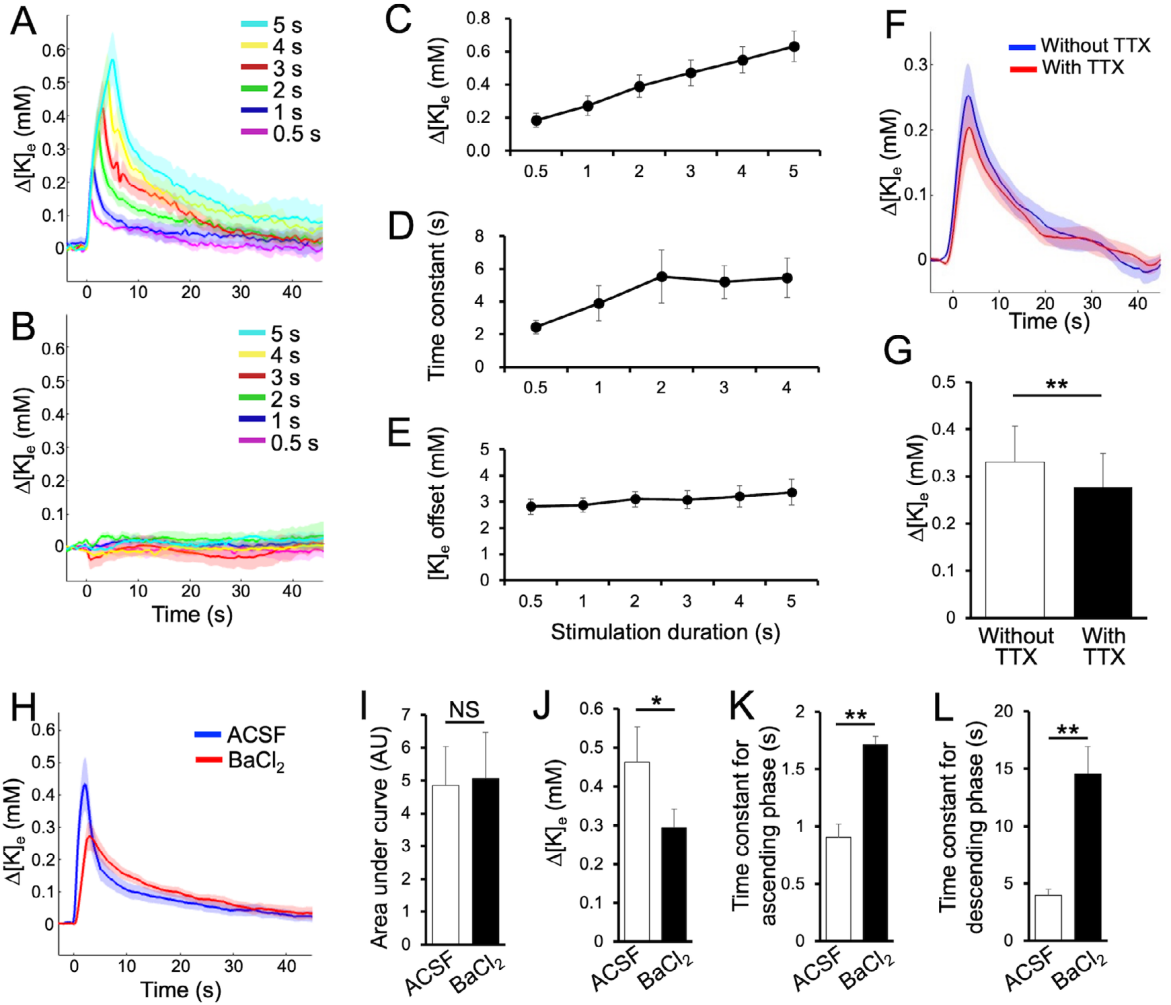
Depolarization releases potassium from the ChR2‐expressing astrocytes in the habenula in vitro. (A, B) Line plots of relative change of the extracellular potassium concentration (Δ[K]_e_) recorded from the lateral habenula of *Dbx1* ^*CreERT2/wt*^; *Rosa* ^*tdTomato/wt*^ with (A) and without tamoxifen at P0 (B) responsive to the 460 nm light applied for 0.5 (purple), 1 (blue), 2 (green), 3 (red), 4 (yellow), and 5 s (light blue). Solid lines and shaded area represent mean ± SEM. (C–E) Line plots of the relative change of Δ[K]_e_ (C), time constant (D) and baseline [K]_e_ before stimulation in each experiment (E) with different stimulation conditions in *Dbx1* ^*CreERT2/wt*^; *Rosa* ^*tdTomato/wt*^ mice with tamoxifen injection at P0. (F) A line plot of Δ[K]_e_ recorded from the lateral habenula of *Dbx1* ^*CreERT2/wt*^; *Rosa* ^*tdTomato/wt*^ with tamoxifen at P0 in response to the 2 s‐photostimulation before (blue) and after tetrodotoxin (red) treatment. (G) A bar graph of the mean peak value in Δ[K]_e_ before (white) and after tetrodotoxin (black). (H) Line plots of the relative change of Δ[K]_e_ in response to the photostimulation before (blue) and after BaCl_2_ application (red). (I–L) Bar graphs of mean area under the curve (I), peak (J), time constant for ascending (K) and descending curve (L) of the Δ[K]_e_ response in ACSF as control (white) and BaCl_2_ group (black). Data are presented as mean ± SEM. *, *p* < 0.05. **, *p* < 0.01. NS, not significant.

Previous studies showed that astrocytes could modulate the neuronal activity by uptake and release of neuroactive molecule such as potassium ion and glutamate in the extracellular space (Eroglu and Barres 2010). To address the mechanism by which photoactivated astrocytes act on neurons in the lateral habenula, we examined the [K^+^]_e_ in the habenula of adult *Dbx1* ^*CreERT/wt*^; *Rosa* ^*ChR2(H134R)‐EYFP/wt*^ mice with or without tamoxifen injection at P0–P2 in vitro. Recordings with a calibrated potassium‐selective microelectrode revealed that 470 nm light application for 0.5 s induced a robust increase in [K^+^]_e_ in the lateral habenula (purple in Figure 5A). We observed a proportional increase in the peak [K^+^]_e_ in accordance with the prolonged duration of photostimulation in *Dbx1* ^*CreERT/wt*^; *Rosa* ^*ChR2(H134R)‐EYFP/wt*^ mice (Figure 5A,C), but not without tamoxifen injection (Figure 5B), suggesting that the effect of light application on [K^+^]_e_ depended on ChR2. The time constant in recovery of [K^+^]_e_ increased when the stimulation duration was elongated from 0.5 to 1.0 s, but seemed to plateau with the stimulation longer than 2 s (Figure 5D). Proportional changes in the peak and time constants could be attributed to possible fluctuations in the [K^+^]_e_ at baseline, as we observed comparable offset levels of [K^+^]_e_ before stimulation, irrespective of the stimulation conditions (Figure 5E) (*F*_5,13.9_ = 0.264, *p* = 0.926, Welch's one‐way ANOVA).

Since we observed an activation of the neurons upon photostimulation, it is likely that both of neurons and astrocytes contributes to the increased [K^+^]_e_ in response to the photostimulation. To examine an origin of the released potassium extracellularly upon photostimulation, we examined the [K^+^]_e_ response to the 2 s‐photostimulation before (blue in Figure 5F) and after tetrodotoxin (TTX) which blocks action potential in neurons (red in Figure 5F). Results showed that TTX suppressed peak response in [K^+^]_e_ to the photostimulation to a minor but significant extent (Figure 5G, *t*_4_ = 4.80, *p* = 0.00866, Cohen's *d* = 2.15, *N* = 5 for each group), while the majority of the response (83.9% of the peak response without TTX) remains even in the presence of TTX, suggesting that the ChR2‐expressing astrocytes are the primary source of the potassium released extracellularly upon photostimulation.

Inward rectifier potassium channel (Kir) sensitive to Ba^2+^ has been implicated in modulation of extracellular potassium ion by astrocytes (Bellot‐Saez et al. 2017). To address its role in ChR2‐expressing habenular astrocytes, we examined photostimulation‐induced potassium release in the lateral habenula before and after BaCl_2_. Although we did not observe significant change in area under curve for total amount of released potassium by photostimulation (Figure 5H,I, *t*_7_ = −0.451, *p* = 0.666, Cohen's *d* = 0.0548, *N* = 8 for each group), Ba^2+^ suppressed the peak value (Figure 5J, *t*_7_ = 2.94, *p* = 0.0218, Cohen's *d* = 0.827, *N* = 8 for each group) and elongated time constant for ascending (Figure 5K, *t*_7_ = −5.33, *p* = 0.00109, Cohen's *d* = 2.91, *N* = 8 for each group) and descending phases of the potassium response (Figure 5L, *t*_7_ = −3.84, *p* = 0.00637, Cohen's *d* = 2.16, *N* = 8 for each group). This suggests that Kir plays a role not only in buffering of [K^+^]_e_ but also in photostimulation‐induced release of the potassium ion in the lateral habenula.

Taken together, these in vitro results suggested that photostimulation of ChR2‐expressing astrocytes activates neurons by releasing potassium ions into the extracellular space.

### 3.5 Photoactivation of the Habenular Astrocyte Induces Depressive‐Like Behaviors

The habenula has been implicated in depressive‐like behaviors based on the finding that the activation and inactivation of neurons by the habenula has prodepressive and antidepressant effects in animal studies (Aizawa et al. 2013). To address whether the manipulation of astrocytes in the habenula leads to the modulation of depressive‐like behavior, we examined the effect of 460 nm light application on the behavior of *Dbx1* ^*CreERT/wt*^; *Rosa* ^*ChR2(H134R)‐EYFP/wt*^ mice expressing ChR2 in the habenula in the open field, tail suspension and sucrose preference tests.

**FIGURE 6:**
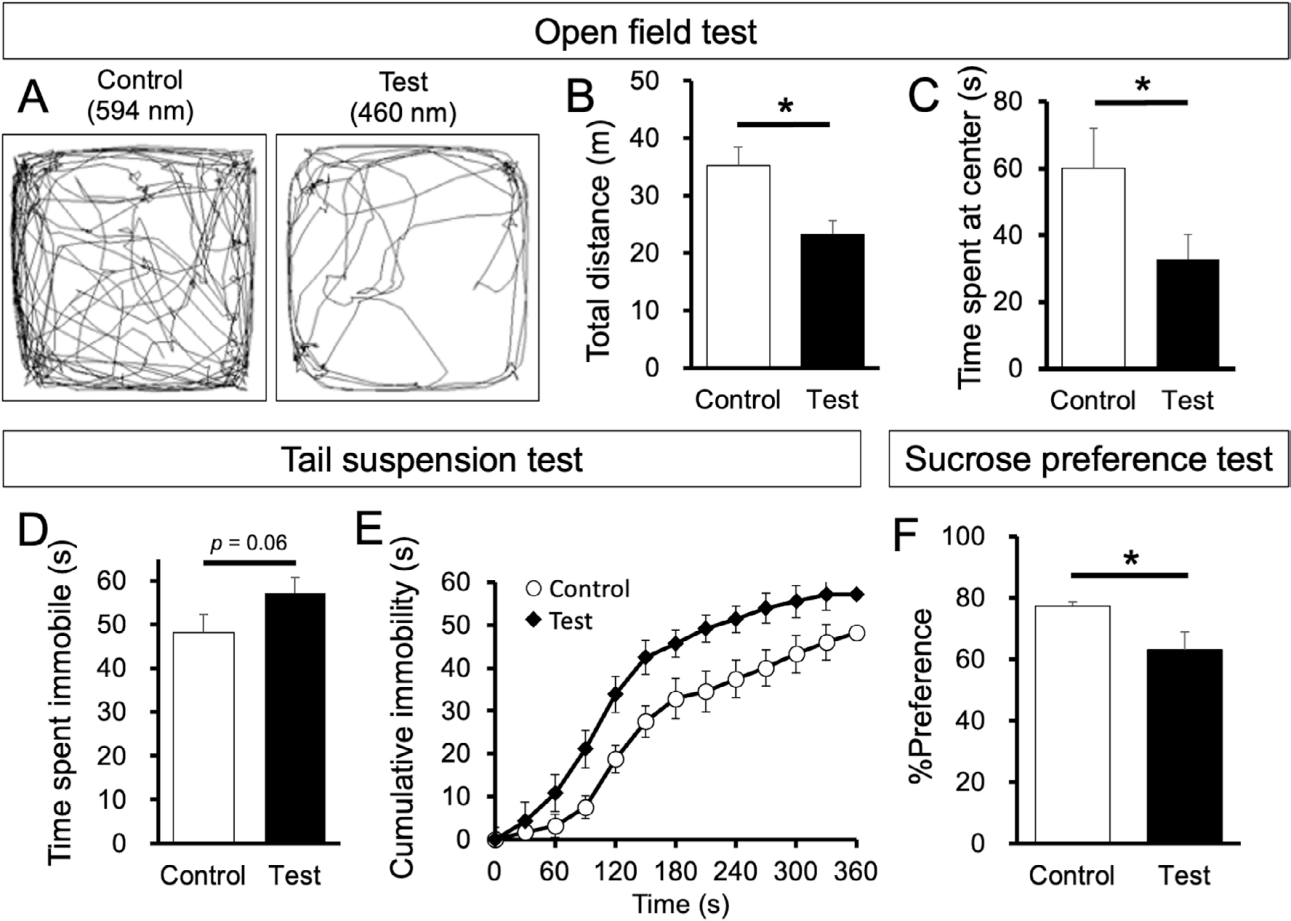
Effect of stimulation of the habenular astrocytes on mouse behavior. (A) Representative traces of activity of *Dbx1* ^*CreERT2/wt*^; *Rosa* ^*tdTomato/wt*^ which received tamoxifen at P0 in the open field arena after photostimulation by 594 nm (Control) and 470 nm light (Test). (B, C) Bar graphs of the mean distance traveled in the whole area (B) and time spent at the center (C) of the open field arena under the control (white) and test conditions (black) during recording of the behaviors for 10 min. (D, E) Graphs of mean time spent immobile (D), cumulative immobile time (E) and mean percentage preference (F) of *Dbx1* ^*CreERT2/wt*^; *Rosa* ^*tdTomato/wt*^ mice that received tamoxifen at P0 under the control (white) and test conditions (black) during tail suspension test (D, E) and sucrose preference test (F). Data in panels B–F are presented as mean ± SEM. *, *p* < 0.05.

We observed that *Dbx1* ^*CreERT/wt*^; *Rosa* ^*ChR2(H134R)‐EYFP/wt*^ mice with photostimulation of the astrocytes in the bilateral habenula with 460 nm light (right in Figure 6A) traveled shorter distances than those with 594 nm light as a control (left in Figure 6A), which was statistically significant (Figure 6B) (*t*_18.2_ = 2.93, *p* = 0.004, Cohen's *d* = 1.25, *N* = 11 mice for each group). Intriguingly, mice exposed to 460 nm light (black in Figure 6C) spent less time at the center of the arena than those exposed to 594 nm light (white in Figure 6C) (*t*_16.7_ = 1.94, *p* = 0.035, Cohen's *d* = 0.825, *N* = 11 mice for each group), suggesting that ChR2 activation of habenular astrocytes with 460 nm reduced thigmotaxis, which is used frequently as a measure for anxiety‐like behavior (Crawley 2007).

In the tail suspension test, mice showed immobility as a despair‐like behavior. Analysis showed that *Dbx1* ^*CreERT/wt*^; *Rosa* ^*ChR2(H134R)‐EYFP/wt*^ mice with photostimulation of the astrocytes in the bilateral habenula (black in Figure 6D) spent a longer time immobile than the control mice (white in Figure 6D) (*t*_15.7_ = −1.62, *p* = 0.063, Cohen's *d* = −0.762, *N* = 9 mice for each group). The difference in immobility between the groups was more obvious in the first 120 s than in the later period of the test, as revealed by analysis of the cumulative immobility time (Figure 6E).

In the sucrose preference test, photostimulation of the habenular astrocytes reduced the sucrose preference as compared to that in control (Figure 6F) (*t*_13.0_ = 2.53, *p* = 0.025, Cohen's *d* = 1.31, *N* = 8 and 7 mice for control and test groups, respectively), suggesting that mice had an anhedonia‐like phenotype by activation of the habenular astrocytes.

Collectively, these results indicated that photostimulation of habenular astrocytes exacerbates depression‐ and anxiety‐like behaviors in mice.

## 4 Discussion

In this study, we found that genetic manipulation of the Dbx1‐expressing domain in the perinatal brain enabled the labeling of astrocytes in the habenula. Through analyses using in vivo and in vitro physiology, we revealed that photostimulation of these astrocytes released potassium, which in turn excited the neurons in the habenula. Behavioral analysis showed that the manipulation of habenular astrocytes caused depressive‐ and anxiety‐like behaviors.

Below, we discuss the mechanisms underlying neuron‐glial interactions in the habenula and their possible involvement in depression.

### 4.1 Developmental Origin of the Astrocytes in the Epithalamus

The epithalamus consists of the habenula, the pineal gland, and the PV thalamic nucleus (Jones 2007). Prosomere 2 in the developing brain specifically expresses the transcription factor Dbx1 in the ventricular zone, from which Brn3a‐expressing neuronal precursors are born (Quina et al. 2009). As Brn3a is essential for the differentiation and survival of habenular neurons (Xiang et al. 1996) and is used as a marker of habenular neurons (Amo et al. 2010), many of these cells are likely to be projection neurons in the habenula. Indeed, the current study corroborated that the neurons born at E13.5 from the Dbx1‐expressing domain gave rise to neurons forming the fasciculus retroflexus and projected to the interpeduncular nucleus. Unlike neurons, only a few studies have examined the origin of astrocytes in the habenula.

The results of the current study are the first, to our knowledge, to show that the Dbx1‐expressing ventricular zone in the developing dorsal diencephalon is a source of habenular astrocytes. Similar to the generation of cortical astrocytes from the ventricular and subventricular zones facing the lateral ventricle (Ge et al. 2012), we found that the Dbx1‐expressing ventricular domain as a source of astrocytes distributed in the epithalamus, such as in the habenula. Intriguingly, these astrocytes migrated laterally but did not cross the lateral borders of the habenula. This may imply a difference in the extracellular environments inside and outside the habenula, such as a repulsive cue from the thalamic structure surrounding the habenula that inhibits further migration. As the current study was limited to cells from the Dbx1‐positive domain, it remains unclear whether other domains in the prosomere 2 contribute to astrocytes in the habenula. Because the subregions of the prosomere 2 are demarcated by differential gene expression, genetic fate‐mapping of astrocytes in other thalamic subregions would clarify their origin in the ventricular zone of the developing diencephalon.

### 4.2 Astrocytic Modulation of the Neuronal Activity in the Habenula

Astrocytes play a pivotal role in modulating neuronal excitability. In particular, clearance of potassium ions from the extracellular space determines neuronal excitability (Rasmussen et al. 2020). Owing to the high permeability of the neuronal cell membrane to potassium ions, a slight increase in the [K^+^]_e_ is likely to induce an increase in spike firing in neurons. Indeed, an experimental increase of the [K^+^]_e_ by 1.0 and 1.5 mM significantly increased neuronal firing in the acute cortical slice in vitro and the awake mouse motor cortex, respectively (Rasmussen et al. 2019). A previous study reported that photostimulation of ChR2‐expressing astrocytes in the striatum increased the [K^+^]_e_ (Octeau et al. 2019) indicating the possibility of modulating motor behavior by astrocytic regulation of potassium ion metabolism. Despite accumulating evidence, the role of astrocytes in other brain regions in the modulation of extracellular potassium ions remains unclear, considering the heterogeneity of astrocytes in the mammalian brain (Yang and Jackson 2019). Indeed, we observed that Kir blocker Ba^2+^ suppressed photostimulation‐induced [K^+^]_e_ increase in the habenular astrocytes, which was not found in the striatal astrocytes. These observations might imply that astrocytes modulate [K^+^]_e_ in a region‐specific manner.

The lateral habenula has been implicated in depression‐like behaviors due to its modulatory function in monoaminergic activity (Aizawa and Zhu 2019). Specifically, a recent study reported that the astrocytic potassium channel Kir4.1 was upregulated in the lateral habenula of rat models of depression, while its loss regulated neuronal firing patterns and had antidepressant effects (Cui et al. 2018). In the current study, we identified the Dbx1‐expressing perinatal domain as the origin of these astrocytes and provided evidence that they have the capability to activate habenular neurons for a prodepressive effect with reduced locomotor activity by increasing the [K^+^]_e_. As judged from a previous report showing that higher [K^+^]_e_ in the mouse cerebral cortex increased locomotor activity (Dietz et al. 2023), the impact of [K^+^]_e_ change on animal behaviors depends on affected neural circuits. Since we attributed habenular astrocytes to the Dbx1‐expressing domain in the perinatal period, developmental defects in the proliferation and differentiation of these developing cells might cause pathological excitability of habenular neurons, leading to high susceptibility to stress and depressive‐like phenotypes.

In addition to potassium ions, glutamate is another important substance released from astrocytes that modulates neuronal activity. Extracellular glutamate, as a determinant of neuronal excitability, is primarily cleared by the astrocytic glutamate transporter, excitatory amino acid transporter 2, and human homologue of murine glutamate transporter 1 (GLT‐1) (Haugeto et al. 1996). Indeed, we reported that astrocyte‐specific knockout of GLT‐1 in the mouse habenula increased susceptibility to acute and chronic stress and induced depression‐like behaviors (Cui et al. 2014). In contrast, photostimulation of ChR2‐expressing astrocytes releases glutamate into the cerebellum (Beppu et al. 2014). Thus, it is possible that the photostimulation of habenular astrocytes modulates neuronal activity, at least in part, by the release of glutamate from stimulated astrocytes. Measurement of extracellular glutamate by genetically encoded biosensors, such as iGluSnFR (Marvin et al. 2013) or enzyme‐based biosensors (Aizawa et al. 2020) would clarify whether photostimulation releases glutamate into the extracellular space from ChR2‐expressing astrocytes in the habenula.

### 4.3 Advantages and Limitations of the Developmental Domain‐Specific Manipulation of Cells

Compared with the genetic manipulation of astrocytes via adeno‐associated viral (AAV) transduction, the current labeling method has an advantage in that it enables the labeling of a subset of astrocytes with a shared developmental origin, independent of anatomical regions. Since the glial acidic fibrillary protein promoter is primarily used in AAV because of its limited genomic capacity, leakage and diffusion of the viral particles are likely to affect off‐target areas anatomically adjacent to the habenula, such as the hippocampus and thalamic nuclei. This reduces the risk of optogenetic manipulation affecting not only the habenula, but also ventral structures, such as the mediodorsal and PV thalamic nuclei, each of which has significant implications for cognitive impairment (Perry, Lomi, and Mitchell 2021) and depressive‐like behaviors (Kasahara et al. 2016), respectively. A lack of functional probe expression, such as that of ChR2, in these off‐target areas would be beneficial for manipulating small structures, such as the habenula.

In the present study, we failed to identify habenular neurons that were specifically derived from the Dbx1‐expressing ventricular zone. A previous study reported that prenatal injection of tamoxifen to *Dbx1* ^*CreERT*^ labeled distinct types of amygdalar neurons depending on the timing of injection (Hirata et al. 2009), suggesting that gene recombination of the reporter by *Dbx1* ^*CreERT*^ occurs in postmitotic progenitor cells. As judged from the labeling of a wide variety of neurons and presumptive glia when tamoxifen was injected at E11.5 in the current study, this might be accounted for by nuclear translocation of CreERT2, at least in part, in neural stem cells. Recently, a novel method was developed to label adult neurons in a birthdate‐specific manner (Hirata et al. 2021). Indeed, *Neurod4* ^*CreER*^ effectively labeled the lateral and medial habenular neurons by injecting tamoxifen at E11.5–12.5 and E13.5–15.5, respectively. Because Neurod4 is a transcription factor expressed transiently in maturing neurons (Tutukova, Tarabykin, and Hernandez‐Miranda 2021), it would be more suitable than the current method for labeling neurons in a birthdate‐specific manner.

The current method had limitations in defining the subregions of the epithalamus. Indeed, the postnatal translocation of Dbx1‐CreERT to the nucleus by tamoxifen‐labeled astrocytes was observed in both the medial and lateral habenula, as well as a part of the PV thalamic nucleus and pretectal thalamic area posterior to the habenula. Based on the wide expression of the reporters, we could not attribute the behavioral alterations to photostimulation of the lateral habenula.

The expression of a reporter, ChR2‐EYFP, was observed in a subregion of the habenula, which could also impair the effect of photostimulation of these cells on behavioral changes. The incomplete coverage of the habenula by Dbx1 domain‐derived astrocytes in the current study could be due, at least in part, to the short action of tamoxifen, which was administered once during the perinatal period. A previous report showed that the half‐life of tamoxifen was 11.2 h at the same dose used in the current study (20 mg/kg) (Reid et al. 2014). Since we observed that a single injection of tamoxifen at P1–P3 resulted in comparable labeling of the cells, daily injections of tamoxifen during P1–P3 in *Dbx1* ^*CreERT/wt*^; *Rosa* ^*ChR2(H134R)‐EYFP/wt*^ mice were likely to increase the number of cells labeled to cover a larger area of the habenula.

## Author Contributions

H.A. designed the study, performed histological and electrophysiological analyses and wrote a draft of the manuscript. M.M., D.K.K., X.Y. and W.T. performed behavioral analyses. L.A.N.O. and F.N. performed potassium recording. T.A. and K.T. provided materials essential to the study. All the authors contributed to the manuscript and approved the submitted version.

## Ethics Statement

All experimental procedures were performed in accordance with the Animal Experiment (approval number A22‐139) and Recombinant DNA Experiment (approval number 2022‐90) plans approved by the Committee of Hiroshima University.

## Conflicts of Interest

The authors declare no conflicts of interest.

## Supporting information


**Figure S1:** A montage of coronal sections of the adult mouse brain to show expression of *Dbx1* ^*CreERT/wt*^; *Rosa* ^*ChR2(H134R)‐EYFP/wt*^.

## Data Availability

The data that support the findings of this study are available from the corresponding author upon reasonable request.
