# Supplementary figures and images for: Potassium Release From the Habenular Astrocytes Induces Depressive‐Like Behaviors in Mice
Source: Glia. 2024 Nov 29;73(4):759–72. doi: 10.1002/glia.24647 (PMC11845841; doi:10.1002/glia.24647)

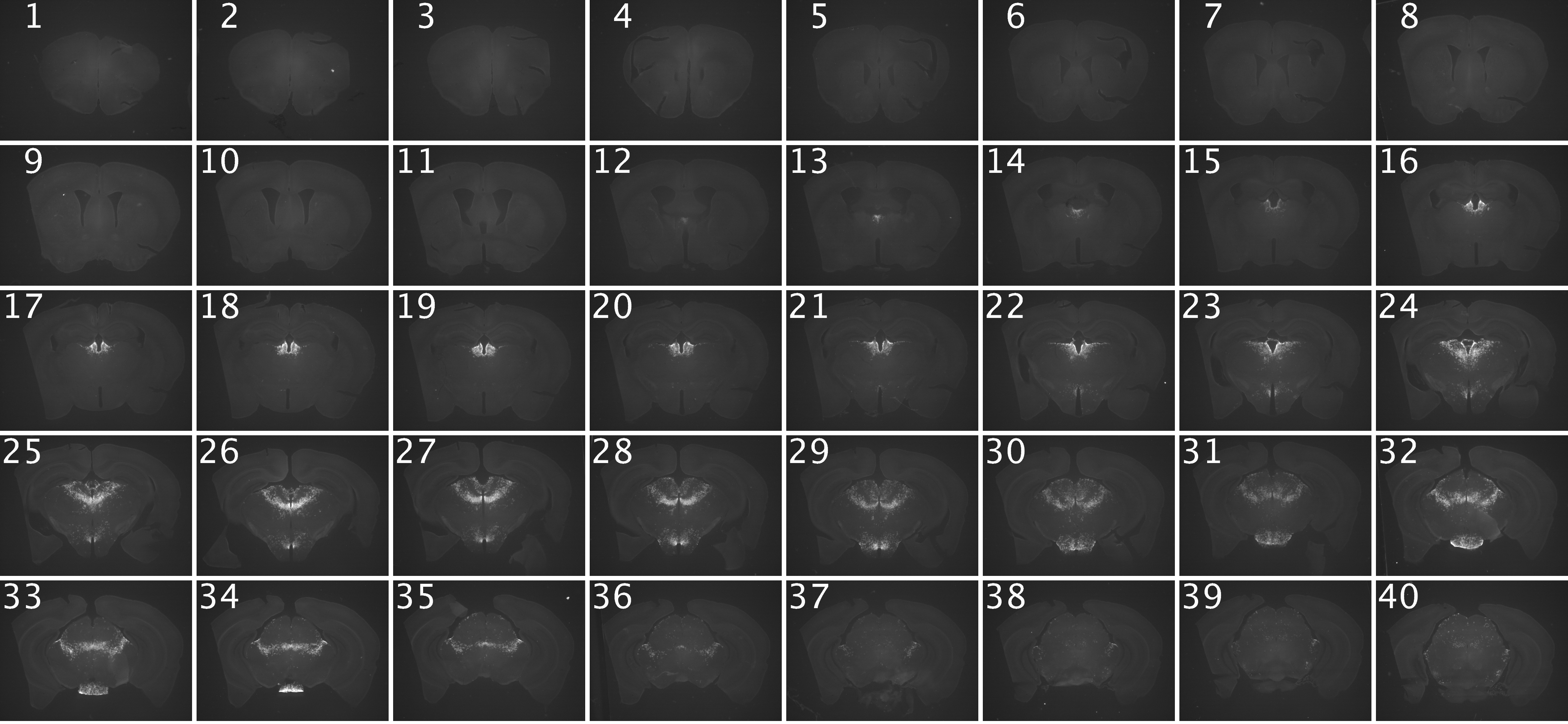

Supplement: Supplementary file 1 — Figure S1: A montage of coronal sections of the adult mouse brain to show expression of Dbx1 CreERT/wt ; Rosa ChR2(H134R)‐EYFP/wt . [file GLIA-73-759-s001.tif]
